# Extracts and Gleanings

**Published:** 1872-04

**Authors:** 


### PART II.

### Abridged Extracts and Gleanings from our Exchanges

*MULTUM IN PARVO.*

Cerebro-Spinal Meningitis.—Dr. C. F. Rodenstein, (Medical Record, March 15, 1872), in speaking of this disease, writes:

Since the beginning of November last I have treated forty cases of cerebro-spinal meningitis. Twenty-nine were well-marked typical cases. The ages of my patients ranged from three to sixteen years. Four died: and one is not yet out of danger. As this epidemic occurred under peculiar circumstances, and presented many features which may be of interest to the profession, I propose to publish soon a full report.

The therapeutics of this disease are notoriously unsatisfactory. The mortality of reported epidemics ranges from thirty to seventy-five per cent. The death-rate of my cases is only ten per cent. But their number is too small to allow any legitimate conclusion to be drawn from such limited statistics as to the value of any particular treatment. I anticipate my report, therefore, by this preliminary statement, that others may not miss an opportunity of testing the value of the remedies I have employed.

At first, I used the tincture of calabar bean, but afterwards I succeeded in procuring the extract. The extract was manufactured by Hazard and Caswell, 2½ drachms of which are equal to two ounces of the bean. I had one drachm rubbed up in glycerine and gave 2–5 drops at a dose.

I administered this drug in every case, as soon as tetanic contractions or apparent paralysis of the sphincter iridis appeared; except in two fatal cases, one of which terminated very rapidly, the other occurred before I had obtained the extract and after the tincture was exhausted.

In the great majority of cases the drug seemed to exert a favorable influence upon the symptoms which it was intended to control. In a few of the milder cases, where there was rather a contracted pupil, I administered atropia in pretty full doses, without, however, influencing the general course of the disease.

In the onset of the attack the symptoms are generally so violent, the skin and mucous membranes so congested, that depletion seems the one thing indicated, and yet I have resisted every temptation to abstract blood. I have neither bled, nor leeched, nor cupped, nor blistered. Ice in bladders to the head and neck mitigated the pain and proved grateful to the patient.

Cathartics I used very moderately, not intending to produce any revulsive effect upon the bowels.

I had no reason to be dissatisfied with this treatment, when afterward, in some of the protracted cases, a prostration set in as extreme as the precedent violence, and a degree of emaciation followed which rivalled the marasmus of the everlasting gastro-intestinal catarrh of starveling foundlings.

In the first days, or whenever, in the course of the disease, there was great restlessness, or violent jactation, I administered moderate doses of chloral hydrate, 10–15 grains, repeated as required. Morphine, I gave only in one case where ice seemed unpleasant and headache was worse.

Although there is a certain rhythm and periodicity in the progress of the disease, I have seen no reason for giving quinine until after convalescence was established, and then, in combination with iron, it doubtless proves beneficial in re-establishing the vigor of the system.

In most of the cases periods of coma follow or alternate with spasms and jactations. In such cases I have found the iodide of potassium of service. But this drug must be administered with a bold and liberal hand. I have given from two scruples to a drachm in twenty-four hours; generally alternating it with the extract of calabar bean, so that my patients received, every two hours, either a dose of calabar (2–5 drops) or a dose of pot. iod. gr. 6–10.

Cerebro-spinal meningitis is not a self-limited disease; it is protean in its manifestations; probably more frightful in its phenomena than severe in its anatomical lesions,—at least in a majority of cases,—and offers, therefore, a hopeful field to the therapeutists, although few victories have as yet been won. To induce physicians to make new trials, I have ventured to state, briefly, the method of treatment I have pursued; so far as its results go, I believe they are more favorable than any that have been obtained.

In general hygiene, little could be done in the present state of our knowledge, as to the cause of the disease. I have used carbolic acid as a disinfectant, and internally, to some extent.

In the February number of THE COMPANION, when noticing the remarks of Prof. N. S. Davis, upon the employment of Calabar Bean, the question was asked, "has it ever been employed in Cerebro-Spinal Meningitis?" Sooner than expected, the answer comes to us in the above. The great fatality of this disease, under every method of treatment yet suggested, should induce the profession to give the calabar bean a fair trial.

Prof. Hammond, asserts that ergot exerts a controlling influence over "spinal congestions." Would not the calabar bean and ergotine, employed subcutaneously, be of service, by their combined influence?

Drugs having the property of relaxing the "tonic spasm" of muscular fibre, like the calabar bean, should, we think, be employed in meningitis. The gelseminum possesses this property to a great extent, and like the calabar bean has, from this very fact, been used for tetanus. It has also been used in cerebro-spinal meningitis.

The Passiflora Incarnata (May-pop), is extolled by Dr. Phares, of Mississippi, both as an anodyne and relaxer of muscular fibre. Its soothing effect upon the nervous system in nervous affections, as neuralgia, etc., he regards as not inferior to opium, while its power of overcoming muscular rigidity has been wonderfully exhibited in his never-failing efforts in curing tetanus in the horse.

These properties, to our mind, point it out as a valuable remedy in Cerebro-Spinal Meningitis, and we hope the profession will give it a trial.

W. T. G.

LAXATIVE PILL.—

℞.—Podophyllin . . . . . grs. v
Pulv. Capsicum . . . . . grs. x
Ex't Hyosciami . . . . grs. xx
Ft. Pills, No. xx.

One taken at bed-time will usually act gently next morning, leaving the bowels loosely inclined.

The following is also an excellent laxative pill—

℞—Soc. Aloes. Pulv. Ipecac,
Ext. Hyosciami . . . . . aa grs. xx
Ft. Pills, No. xx.

On taken at bed-time will be followed by a mild, painless semi-solid evacuation next morning.

R. C. WORD, M. D.

Tunnel Hill, Georgia.

Bromide Potassium in Spermatorrhea.—I am induced to report the following case, not from anything *unique* in the symptoms or the treatment, but merely as a contribution to the literature of the subject. On the 14th of January, 1871, Mr. ——, an intelligent and fine looking young farmer of 23 years, consulted me for involuntary nocturnal emissions. My patient confessed that he had been guilty of masturbation for many years past, "but never indulged in it to excess;" had left it off entirely for the past six months. For the past four months he has had from three to five emissions a week. Three weeks ago he visited the city of ——, spent the night with one of the demi-monde, and then for the first time discovered that he was "entirely impotent;" and to add to his troubles he is to be married the latter part of March. I think I never saw a man more profoundly dejected. Upon examination the genital organs present a normal appearance. Upon passing a No. 10 steel sound, slight tenderness is manifested in the prostrate portion of the urethra. After explaining to my patient that his case was by no means so bad as he thought it, that his complaint, so far from being an unusual one—as he thought it—was very common and usually amenable to medical treatment. I directed that he should take bromide potassium—thirty grains in an ounce of water, upon going to bed. He left my office, much cheered, with directions to report in three days.

17th.—Patient more profoundly dejected, if possible, than when first seen. He had taken the medicine as directed; the emission failed to occur the first night, but has been as bad as ever for the past two nights; expresses himself as feeling certain that there is no cure for him. Encouraged to persevere, and directed to take the same dose in the morning as at night.

22d.—Patient much brighter and more hopeful, "but does not want to whoop before he gets out of the woods;" has had but two emissions since last seen, on the first and fourth nights; to continue the bromide.

I was summoned to the bedside of a dear relative, residing in a different part of the State, and consequently did not see my patient again for the space of two weeks. In that time "a change had come o'er the spirit of his dreams." He had taken the bromide regularly as directed, and had passed from a state of deep melancholy to be the bright, vivacious man he had formerly been. He was now directed to leave off the bromide in the morning, but to continue it on going to bed. This course was continued for several weeks longer, when the patient was discharged, cured. My patient was married in March. He informed me, within the past week, that he has never been troubled with a recurrence of the emissions since I discharged him. This is a typical case of several that have occurred in my practice, and I can always rely confidently upon the curative action of bromide potassium in spermattorrhœa, when uncomplicated by organic lesion.

*Lexington, Ga.* FOSTER, M. D.

REMARKS ON OLD REMEDIES.—Regarding it the duty, especially of those who have been harnessed for long years to the car of practical medicine, to contribute, however little it may be, to the general stock of knowledge, I would call the attention of the younger members of the profession to the employment of copious injections of the chloride of soda in chronic dysentery. Amid the multiplicity of remedies too often ushered forth with high praises, the physician is often at a loss what course to pursue. Some six months ago I was called to see a negro man, aged about forty, who had for many months been afflicted with bloody, mucous passages. He was much reduced in flesh, skin dry and swarthy, expression that of suffering, eyes appeared somewhat jaundiced, appetite feeble, abdominal wall was depressed, extreme anxiety. Living in what was called a malarial or swampy locality. He had been well dosed with mercury, had taken quinine and opium, been subjected to the saline treatment with and without laudanum—to turpentine with paregoric and sweet spirits nitre—to bismuth, with astringents, etc.; yet he was not well.

He was ordered a pill every night of two grains of blue mass, 1 grain ext. hyoscyamus, $\frac{1}{6}$ grain podophyllin. He took the pill for a week, and I am satisfied with benefit, but in a day or two we commenced with the injectiens of the chloride of soda (long ago recommended) of the strength of one-half ounce to a pint of cold water, with instructions to throw up before stopping a gallon of the mixture. In a few days the strength of the injection was doubled, so that it should contain in 10 parts of water one part of the chloride. His food was limited to crackers and stale bread and as much of the fat of sweet corned or pickled pork, broiled crisp on coals, as he wished, or at first could be induced to eat. The injections were to be repeated daily. He commenced early to improve, and in about two months his disease was gone. In the chronic form of this disease, as well as of diarrhœa—a still more fatal affection—the bowels seem to collapse, their coats to stick together, and until distended, there is no hope of cure. Copious injections may suffice to distend the partially collapsed, and, though inflamed, debilitated colon. So in chronic diarrhœa—particularly of children—farinaceous substances, and, especially in the latter part of summer and fall, the first dug sweet potatoes may be eaten with immediate benefit. I have seen many children saved from death, (in extreme emaciation), by their use. The immoderate use (filling up the intestines) of the roasted potato, before it has become candied, is a poison in the disease.

Permit me to endorse and recommend to the profession, the employment of the permangate of potassium in injection, of the strength of from one to three grains to the ounce of water in chronic lucorrhœa or whites. It has so happened to me that it has succeeded in many cases that rebelled against all other remedies.

THOMAS A. COOKE,

Washington, St. Landry, Louisiana.

PUERPERAL CONVULSIONS.—Mrs. T——, age 26 years, was delivered of her third child in August, 1871, by a midwife. Everything was supposed to be right, and after the usual directions her nurse left her. On the second day after delivery, she complained of fullness and tension in the pelvis, and some pain and uneasiness over the hypogastric region, the lochia ceased, and fever begun to appear. The midwife was sent for, and gave her a variety of teas, and applied warm fomentations to the abdomen, which had continued to grow in tenderness and tension; soon after midnight, the fever increasing, her head began to ache, and restlessness supervened. About midnight, convulsions made their appearance. These increased in violence until about 10 o'clock, A. M., of the third day after delivery, when I saw her. I found her complaining with her head and left shoulder; her arm could scarcely be moved without great pain; skin very hot and dry; pulse 130, full and resisting; lochia suppressed; abomen full and somewhat tender; conjunctiva injected; eyes vacant and wild, and the convulsions occurring at intervals of about forty minutes.

I bled, took about sixteen ounces, and gave the following:

℞—Sub. Mur. Hydg. Mit. . . . grs. x,

Sulph. Quinna, . . . . . grs. xii.

Applied cold applications to the head, and used a vaginal injection, composed of sweet milk with aqua ammonia, fifteen drops to half ounce of milk, repeated at intervals of two hours. She had but two convulsions after the bleeding, and the compound doses as given above, and in two hours her pulse had gone down to ninety per minute; she was calm; slept some, and was perspiring. I repeated the quinine at intervals of two hours, in four grain doses. Her bowels were well evacuated during the day. I continued the quinine for twelve hours, when perspiration becoming too profuse and exhausting, I omitted it, and gave muriated tincture of iron, under which she recovered, not, however, until after the supervention of a slight hectic fever, which lasted for twelve days.

I mention this case more particularly to show the sedative power of quinine, which it will invariably have when following venesection, as also the metastasis of inflammatory action from the pelvic peritoneum to the shoulder and head, drawn hither, no doubt, by mammary excitement, producing, in its course, not only arthritis of the left shoulder, but was rapidly developing arachnitis, for I think this case, in its incipiency, was puerperal peritonitis.

In all such cases the lancet should be used; it cannot be arrested without risk to the patient, but it will not do to rely on venesection alone. We must have a sedative, and prominent among these is quinine.

My first case of eclampsia was well marked—a primipara. I relied on the lancet alone—bled three times, but my patient expired.

My second case was also well marked—a primipara. I bled and used chloroform as a sedative, by inhalation. The convulsions ceased, but the skin remained dry, head hot, together with restlessness and jactitation, and it became necessary to continue the use of it to prevent a return of the convulsions, which I did, at intervals for twelve hours.

My third case was also well marked—a primipara. I used the lancet, and then quinine as a sedative. It acted like a charm, and has never failed me since in any case of the kind. It might relieve without the lancet, but the urgency and terror of eclampsia is such that I think it ought never to be omitted, especially when the habit is plethoric. The dose, at the outset, should be large (from twelve to twenty grains).

I would state, in this connection, that in every case which has come under my observation, (of the primipara) the uterus has seemed to take on a tetanic condition, remaining fixed and tense for hours. In view of this symptom, I have been in the habit of using hot fomentations to the abdomen, and in some instances it has been five or six hours before there were any signs of motion or relaxation in the uterus. I have never used the atropia, but think, in such cases as a local application to the os tincæ, it might be in place.

In our country, eclampsia has not occurred oftener than once in every one hundred parturients, and two-thirds of them are with the primipara. GEORGE F. TAYLOR, M .D.

LaFayette, Alabama.

EFFECTS OF BROMIDE OF POTASSIUM.—Dr. Julius Levy, of Berlin, writes that if bromide of potassium, in drachm doses, three times daily, is continued for months, a series of boils will be apt to be produced. He says if some preparation of cinchona be given with the bromide, no boils or other evil sequelæ will arise.—*Med. Rec'd.*

A Plan for Facilitating the Reduction of Strangulated Inguinal Hernia by Taxis.—A correspondent of the British Medical Journal, calls attention to a method recommended by Sentin. The patient is to be placed on the back, with the hips well raised, and in case the hernial tumor is not reducible by the ordinary manipulation, the surgeon is to pass his index finger up into the ring, if possible, to the outer side of the gut. After crowding the point of the finger past the column of the ring, strong and constant pressure is made, until the fibres of the ligament give way, or the opening is perceptibly enlarged, when it will be found that the intestine can be returned easily to the abdomen by the usual effort. The conditions which contraindicate a resort to this procedure are:

1. The fact of the hernia being old and irreducible.
2. The presence of the constriction at the inner ring—the external ring being too small, and the canal too long, to admit the finger.
3. The existence of general symptoms of grangrene of the intestine.

Flatulent Dyspepsia.—The late Dr. Trousseau, of Paris, (New Sydenham Society's Translation), in this form of dyspepsia, administered alkaline preparations for a few consecutive days, followd by the administration of bitters. For five or six days, at the beginning of the two principal meals, and on retiring at night, the following powder is useful: Magnesia, chalk, bicarbonate of soda, four to six grains each, mixed immediately before taken, in a fourth of a tumbler of water. This to be followed by the employment of quassia. In the morning fasting, and at mid-day, at an equal interval between the two principal meals, the patient ought to drink a cup of the infusion of quassia, prepared by leaving a teacupful of cold water for fifteen or twenty minutes, in a goblet made of this wood; or, which is much better, by macerating two grammes of quassia shavings in cold water from four to six hours. In these cases wine of cinchona is also indicated. It ought to be given either immediately after meals, or immediately after the patient has taken a small quantity of food, in order to prevent the pain in the stomach which is apt to be excited when taken fasting.

Digitalis.—*Its Action on the Heart.*—Digitalis used to be considered a cardiac sedative because it was found to relieve palpation, which was thought to be over-action of the heart. It is now known, however, that there is no such thing as over-action, that hypertrophy is rather evidence of deficiency than of excess of power. It consequently follows, that if digitalis relieves palpitation it must be by a tonic action on the heart. It is in cases of deficient expulsive power of the heart walls that digitalis is chiefly of use; where the heart is distended, and in contraction, only gets rid of a little blood off the top, remaining more or less full in systole. Under digitalis the contraction of the ventricles becomes complete, and the pulse consequently steadier, firmer, and less compressible, the system is relieved, dyspnœa is abated, the deficient secretion of urine is improved, and free secretion takes its place. Dropsy is thus often relieved. (Dr. J. M. Fothergill, British Medical Journal, July 8, p. 27.—*Braithwaite's Retrospect.*

THE USE OF DIGITALIS.—For persistant use digitalis should be given in powder. A favorite pill is the following: Half a grain to a grain of powdered digitalis, with an equal quantity of the dried sulphate of iron in powder, and a morsel of cayenne in extract of gentian or aloes and myrrh pill. Thus we secure, at one cast, an action on the circulation, the addition of iron in a form which will act locally on the stomach, and thus act as an astringent on the gastric catarrh so common among the sufferers from heart disease. (Dr. J. M. Fothergill, British Medical Journal, August 5, p. 148.—*Braithwaite's Retrospect.*

VARICOSE VEINS.—*Application of Strong Nitric Acid.*—Varicose tumors may be successfully treated as follows: Suppose, for example, that the tumor is on the leg. Pressure having been made above and below the tumor, the intuguments must be raised from it, and incision by transfixion made in the skin so as to expose the tumor. The fuming nitric acid is then to be applied to the external coat of the vein. No pain will attend this application. On the following day the contents of the tumor will appear solidified, and the acid may be applied again. The process of solidification will then go on rapidly, the tumor, at the same time, decreasing in size. In the course of a week a small slough will come away, leaving a wound which may be dressed with tinct. benzoin co. and glycerine. This practice was suggested by Sir Dominic Corrigan, to whom the idea occurred from the success which generally attends the treatment of hemorrhoidal tumors by nitric acid.—*Braithwaite's Retrospect.*

ULCERATED THROAT AND CAVITY IN THE LUNG—*Inhalation of Iodine.*—The readiest and most effectual means of applying this practice is to use a solution of twenty grains of iodine to the ounce of hydride of amyl (a solution of great service in other cases), and to dilute a portion of this with more of the hydride until the vapor of the iodine given is scarcely at all irritating to the throat. This solution may be inhaled from a little funnel of parchment paper, holding in it some finely-teazed cotton-wood, on which the solution may be dropped. It should be held a little way from the nostrils and mouth, so as to allow the admission of plenty of fresh air. The quantity of solution used should be so regulated that about five grains of iodine are inhaled at a time.—*Braithwaite's Retrospect.*

DIABETIS.—*Ammonio-Saline Treatment.*—It has been found, by analyses of diabetic blood, that there is a great deficiency of certain alkaline salts. These salts are absolutely necessary in order that the sugar which is formed in disease, just as in health, should be burnt off at the lungs. M. Mialhe, who discovered the above fact, considers this deficiency the primary cause of the diabetes. Whether this is so or not, there is no doubt that such deficiency must react upon the disease. Accordingly, treatment directed to supply this deficiency is likely to prove of service, and in actual practice such is found to be the case. The best saline mixture is composed of carbonate of ammonia, ten grains; phosphate of ammonia, ten grains; carbonate of soda, ten grains; tincture of ginger, a few drops; three times a day in an ounce of water. This mixture is very grateful to the patient, it relieves thirst, and mitigates the morbid appetite. The tongue generally becomes moist, the urine diminishes in quantity, and contains less sugar. In one case which may be taken as an average one, the amount of sugar was reduced from thirty grains to the ounce of urine, to six grains, and the amount of urine from fourteen to four pints.—*Braithwaite's Retrospect.*

DIET IN DIABETES.—Dr. Wadham, of St. George's Hospital, has made a most exhaustive series of experiments, to determine the relative influence of bread, honey, and sugar, upon the amount of urea and sugar excreted in diabetes. He finds that bread, in all cases, and in every stage of diabetes, largely increases the amount of urine, urea, and sugar secreted, and in every way aggravates the symptoms of the disease. Honey, on the other hand, may often be advantageously used as an article of diet, because in some cases a large amount of it may be eaten without materially increasing the weight of urea or sugar excreted, and the weight of urea may even diminish. Pure white sugar may be added to the diet in diabetes, with every prospect of a beneficial result, for its use is accompanied by a dimunition in the amount of urea excreted, and, when given in large quantities, less than one-sixth of the amount escapes as sugar in the urine, the remainder being either burnt off or otherwise appropriated to the use of the system.—*Braithwaite's Retrospect.*

SUPPURATING SORES AND WOUNDS.—*Mode of Applying Iodine to.*—Iodine dissolves readily in hydride of amyl, and when this solution is applied to the skin, the volatile hydride passes off and leaves the iodine behind, stranded on the part in most equal form of distribution. This application is of singular utility in cases of hard open sores, where it is desired to apply iodine evenly and deeply. Thus, in cases of open strumous glandular disease, the solution plays an important part as a means of cure, and the same in chronic indolent bubo. In bad sloughing fœtid ulcerative and suppurative wounds, and in cancer, no solution is so simple, painless and effective. In these last-named cases it exerts more than a curative influence, it destroys decomposing organic products. The solution may be gently poured over the parts. There is no necessity either for cotton-wool or brush. (Dr. B. W. Richardson.—*Braithwaite's Retrospect.*

INCOMPATIBLES WITH THE PERCHLORIDE OF IRON.——M. Bouilhon (New Remedies, translation from L'Union Pharm.), gives the subjoined list of incompatibles: Salts of silver; protosalts of mercury; alkalies, their carbonates and bicarbonates; the arsenites and arseniates; borate of soda; tannin and vegetable astringents: gums; vegetable extracts, and vegetable infusions; albumen; casein.

AN EFFICIENT HÆMATIC.—Dr. Humphrey Peak, of Vasalia, California, publishes in the Pacific Medical and Surgical Journal, the following formula for a pill which he has used with signal benefit, for the past ten years :

R.—Quiniæ Sulphatis . . . . . oz. j,
Ferri Redacti . . . . . . oz. jss,
Strychnia, acidi arseniosi, . . aa grs. iij,
Confectionis rosorum, vel. mucilaginis acaciæ, q. s. ut ft. pil. lx.

He says; "The range of morbid conditions to which this pill is applicable, is astonishing to any but the educated of the medical profession. It is applicable to all cases—saving, perhaps, organic disease of important organs; and here, indeed, it could do no harm, although it might be impossible to cure—when the object is to improve the quality of the blood. But it is more particularly applicable and useful, and *curative*, in the whole list of what I take the liberty of calling *malarial, cachexiæ,*

Delirium Tremens.—Dr. C. W. Earle, of Chicago, (Chicago Medicel Examiner, April 1st, 1872), in writing of this disease, remarks:

At least two indications present themselves for treatment in the majority of patients admitted to our Home, viz: to give nourishment and procure sleep. The first is best met by giving beef tea, milk and eggs, etc., etc.—in a word, concentrated nutriments which are easily digested. The stomach is not in a condition to digest solid food, and we therefore give the liquid nourishment. To quiet the extreme nervousness during the day, previous to giving chloral, we are in the habit of giving bromide of calcium, bromide of potassium, valerian, etc. A combination of the bromides of calcium and potassium with cannabis indica is excellent. But surpassing them all, when night comes and we wish to give our patient sleep, is the chloral. It matters little how it is given. It is not as necessary in the cases we treat at the Home to disguise the taste of the drug as in paivate practice. The sharp, pungent taste, to one habituated to the use of *ordinary whisky*, is not particularly unpleasant. Fifteen grain doses repeated often, until the required result is obtained, seem to give a better effect than to give larger doses.

The exhibition of morphine with chloral seems, in some cases, to give a better result than either drug given singly. The following formula, of which a teaspoonful may be given every two hours, has given decided benefit:

R.—Chloral Hydrate . . . . . dr. ii.
Morphiæ Acet. . . . . . . grs. ii.
Syrupi Zinziberis . . . . . oz. ii.—M.

Patients on the verge of delirium taking this for ten or twelve hours, and followed with somewhat larger doses of chloral, very frequently escape the threatened attack, and make a rapid recovery.

Antidote to Liquor Poisoning.—The physician in charge of the drunkard's department of Blackwell's Island, says that milk or eggs are antidotes for liquor poisons, and those who must drink liquor, should mix milk or eggs with their "poison," which will take effect on those articles instead of the toper's stomach.

The steam from a mixture of an ounce of aqua ammonia to a quart of water, inhaled once a day, is said, by Mr. John Grantham, (British Med. and Surg. Journal), to relieve and even cure attacks of whooping-cough.

New Mode of Administering Copaiba.—Dr. J. H. Wehner (*Medical and Surgical Reporter*, Feb. 17, 1872) says: In chronic cases of gonorrhœa I have obtained the best therapeutical action of copaiba with the entire absence of its nauseating and other disagreeable effects from the administration of that drug, combined with opium in the form of a rectal suppository. The following formula I have adopted, as the suppositories made therefrom are perfectly unobjectionable:

R.—Copaibæ, . . . . fl oz. vi.
Opii pulv., . . . . gr. vj.
Olei theobromæ,
Cetacei, . . . . . aa. oz. jss.
Cereæ albæ, . . . . gr. xlv.—M.
Misce secundum artem et fiant suppositoria, No. xij.
Signa—One to be introduced into the bowel morning and night.

If constipation occurs it may be readily overcome by a moderate dose of Rochelle salts.

The very encouraging results which I have had induced me to make this communication.

Stimulating Hypodermic Injections in Typhoid Fever.—We find in the *Gazette Hebdomaire* of June 16th, some account of a novel mode of treatment adopted by a German physician in the collapse of typhoid fever. The subjects were Prussian soldiers who, during the siege of Paris, suffered from typhoid of extremely ataxic type, with feeble heart-movement, small and irregular pulse, cyanosis, cold extremities, and general collapse. Dr. Zuelzer, observing the resemblance to the choleraic condition, in which he had derived good results from stimulating hypodermic injections, determined to try the same method, and accordingly injected into each of the four extremities ten drops sulphuric ether, with five drops "anisated, ammoniacal solution." "The results were remarkable. The pulse, before small and irregular, became full and strong; the contractions of the heart, which had been feeble and irregular, became energetic and regular; its impulse, before imperceptible, was now well marked. Frequently, after one or two injections, the cyanosis and collapse vanished. This plan has the additional advantage of gaining time for other treatment. Small abscesses are formed at the places of puncture, but they are of no importance."

### TRREATMENT OF HEADACHE.

Dr. Bradnack (Buffalo Medical and Surgical Journal, Jan. '72) says:

The treatment of this malady divides itself naturally into two parts: first, that proper during the attack; second, that appropriate in the internal. I believe we possess remedies capable, in most instances, of entirely, or nearly entirely, allaying the pain during the attack; and others administered during the intervals of effecting in a few months a permanent cure. To say that I have wholly originated this method would be to make a strong claim; but, that it has never in this disease been systematically employed before, I am certain.

In the proposed treatment of this disease, we, of course, adhere to general principles. If there exist complications, whether or no they assumed to act as exciting causes, they should be removed, or, so far as possible, paliated. If there be constipation, the usual treatment for constipation is indicated. If examination reveals the existence of a uterine displacement, it should be remedied. If tobaccos are used in excess, they must be either discontinued or used in moderation. Suppose the treatment to be commenced the day after an attack of headache. Assuming the non-existence of any important physical lesion I find it advantageous, provided there are no contraindications, to begin by the administration at night cf one or two of the following pills which, during the entire course of treatment (say six months), may be given one in three weeks:

R.—Mass hyd.,
Ext. coloc. com.,
Pulv. aloes soc., . . . aa. sc.j.
Pulv. ipecac., . . . . gr.vj.—M.
Ft.—pil., no. xij.

This pill to be followed in the morning by one drachm of sulphate of magnesia.

As a permanent medicine, I then prescribe three drops of liquor potassæ arsenitis, to be taken in one drachm of water after each meal, for certainly three, and usually six months, its use being suspended one day every three weeks when the above pill is taken.

If the patient be delicate, and complains much of coldness of the extremities during the attacks, and frequent chilliness during the intervals, the following prescription is substituted for the liquor potassæ arsenitis:

R.—Liq. arsenicalis hydrochlor., . dr.ss.
Quiniæ disulphat., . . . gr.xij.
Liq. ferri perchlorid, . . dr.ij.
Aquæ. . . . . . f.dr.vj.—M.

S.—One tablespoonful in a wine glassful of water, twice a day, after meals.

When an attack of headache begins I adopt the following plan, with minor modifications according to existing circumstances and complications. I direct the patient to sit in an easy chair (avoiding an incumbent position, as tending to cerebral congestion by means of gravitation), and to place his or her feet in a hot bath of mustardized water, the hands also in a similar hot bath, minus the mustard; and if it can be tolerated (though females frequently cannot tolerate it) a bag of pounded ice to be placed upon the head, covering as much as possible of the occipital region, and thereby bringing decongestive influence to bear upon the cerebellum and the medulla oblongata. These accessory measures to be followed by a dose of the following medicine:

R.—Potassii bromid., . . . dr.vj.
Ammon. bromid., . . . dr.ij.
Potassii iodidi., . . . gr.vj
Infus columbæ, . . . f oz.ix.—M.

S.—One desert spoonful in an ounce of water.

One or two doses of this prescription will usually suffice either to very greatly palliate or else entirely relieve the most distressing and agonizing headache, provided only it belongs to the class under consideration. But, to produce the best effects, this remedy should be administered as early as possible after the commencement of the attack. In some cases patients experience prodromic symtoms. When these occur, the threatened attack may often be rendered abortive by the timely administration of the medicine.

---

Sulphocarbolate of Zinc in Otorrhœa.—At a recent congress of German surgeons in Prague, Dr. Zaufal said that he had used a solution of the sulphocarbolate of zinc in fourteen cases of otorrhœa, with satisfactory results. The strength of the solution was one or two grains to the ounce.—*Medical and Surgical Reporter.*

### ON THE UTILITY OF CALOMEL IN INFANTILE INTESTINAL AFFECTIONS.

Dr. E. P. Hurd, (Boston Medical and Surgical Journal, December, 1871), writes:

I believe that in the present state of infantile therapeutics, we cannot profitably dispense with calomel in the treatment of the gastro-intestinal complaints of childhood. Much as we may deprecate the indiscriminate use of mercurials, and much as we may theoretically condemn their exhibition altogether, cases will continually occur, in which we shall find ourselves compelled to resort to some preparation of the greatly abused hydrargyrum.

The following cases, selected from many similar ones in my portfolio, will illustrate what I have said:

Case I.—Mary G., æt. two years, had been suffering for a fortnight from an affection of the stomach and bowels, aggravated by teething, as she was cutting two of her molar teeth. When I first saw her, on the 8th of November, the prominent symptoms were obstinate and uncontrollable vomiting, with constipation, great restlessness and prostration. Bowels had been confined for several days: abdomen swollen and hard; no particular head symptoms; tongue moist, with cream-colored fur; much thirst, but drinks were instantly rejected; frequent retching, even when food and drink were withheld. Occasional febrile attacks, followed by profuse sweats.

Here, said I, is a case where I used to give calomel, but this child shall get none of it.

I tried a dozen things. The simple herb teas, mint and anise, with magnesia; enemata to promote a soluble state of the bowels. It was of no avail; the herb teas "did not stay down a minute," the injections "came back." Bicarb. soda was tried, with bismuth, and this failing, minute doses of opium ground with bismuth and white sugar. I waited a few hours and returned. The powders had all been vomited as soon as taken. I took a hint from a favorite eclectic journal, and prescribed ipecac, one-tenth of a drop of fluid extract to be taken every two hours, alternately with one-fourth of a drop of the tincture of veratrum viride. Again I was foiled. I increased the quantity of ipecac to half a drop, then a drop of Tilden's Extract, but to no effect. Then I administered podophyllin, one-twelfth of a grain, rubbed up with sugar of milk. This surely *must* relieve, I thought. Four powders were given, at intervals of two hours, not one of which was retained. The child was taking milk and lime-water for nourishment, but little of which, however, remained on the stomach. The case was becoming desperate, and bid fair to pass into the hands of another physician.

"There is one thing," I said, "which, antiquated as it is, and though it is passing out of fashion, does not generally so completely fail me. Now for the submuriate." I prescribed as follows:

R.—Hydrarg. Chlorid. Mitis, . . gr. x,
Magnesiæ ustæ, . . . gr. xx.—M

Ft. chartæ No. x. S. One powder every two hours.

I ordered the milk and lime-water, of which she had been taking a tablespoonful every hour, to be omitted, and corn coffee to be substituted, to be given *ad libitum*, as the child was not disposed to drink much. A tansy bag, which had been dipped in warm vinegar and over the surface of which a little mustard had been sprinkled, was applied over the stomach. The swollen gums were freely lanced.

The next morning I was delighted to find my little patient better. The powders had all been given, and not one had been rejected; there had, in fact, been no vomiting during the night. The child had slept more than half the time. Much corn coffee had been taken. There had been several dark, fœtid, bilious discharges. I need not dwell further on this case. Suffice it that there was rapid improvement from that date.

---

### TO REMOVE TAR, PITCH, TURPENTINE, ETC.

Dr. D. D. Binkerd, (Medical and Surgical Reporter, February 3d, 1872), writes:

By accident, I recently discovered a simple combination that will speedily and effectually remove from glass, porcelain, hands, or any part of the body, Venice turpentine, tar, pitch, or any sticky substance of a like nature that will resist warm water and soap. It is entirely harmless to the skin, and yet it will remove these substances as promptly and as thoroughly as soap and water will remove common dirt.

*For cleaning Glass.*—An amalgam of the pulverized extract of licorice and oil of aniseed. This seems to combine with the turpentine, and it may then be rubbed dry and clean with a pledget of cotton.

For cleansing tar or pitch from the skin, make the mixture about the consistency of thick cream, and rub on thoroughly with the hand; then follow with a piece of good soap, a sponge and warm, soft water.

